# Expression of an IKKγ Splice Variant Determines IRF3 and Canonical NF-κB Pathway Utilization in ssRNA Virus Infection

**DOI:** 10.1371/journal.pone.0008079

**Published:** 2009-11-26

**Authors:** Ping Liu, Muping Lu, Bing Tian, Kui Li, Roberto P. Garofalo, Deborah Prusak, Thomas G. Wood, Allan R. Brasier

**Affiliations:** 1 Department of Medicine, University of Texas Medical Branch (UTMB), Galveston, Texas, United States of America; 2 Department of Molecular Sciences, University of Tennessee Health Science Center, Memphis, Tennessee, United States of America; 3 Department of Pediatrics, University of Texas Medical Branch (UTMB), Galveston, Texas, United States of America; 4 Sealy Center for Molecular Medicine, University of Texas Medical Branch (UTMB), Galveston, Texas, United States of America; 5 Department of Biochemistry and Molecular Biology, University of Texas Medical Branch (UTMB), Galveston, Texas, United States of America; University of Hong Kong, Hong Kong

## Abstract

Single stranded RNA (ssRNA) virus infection activates the retinoic acid inducible gene I (RIG-I)- mitochondrial antiviral signaling (MAVS) complex, a complex that coordinates the host innate immune response via the NF-κB and IRF3 pathways. Recent work has shown that the IκB kinase (IKK)γ scaffolding protein is the final common adapter protein required by RIG-I·MAVS to activate divergent rate-limiting kinases downstream controlling the NF-κB and IRF3 pathways. Previously we discovered a ubiquitous IKKγ splice-variant, IKKγΔ, that exhibits distinct signaling properties.

**Methodology/Principal Findings:**

We examined the regulation and function of IKKγ splice forms in response to ssRNA virus infection, a condition that preferentially induces full length IKKγ-WT mRNA expression. In IKKγΔ-expressing cells, we found increased viral translation and cytopathic effect compared to those expressing full length IKKγ-WT. IKKγΔ fails to support viral-induced IRF3 activation in response to ssRNA infections; consequently type I IFN production and the induction of anti-viral interferon stimulated genes (ISGs) are significantly attenuated. By contrast, ectopic RIG-I·MAVS or TNFα-induced canonical NF-κB activation is preserved in IKKγΔ expressing cells. Increasing relative levels of IKKγ-WT to IKKγΔ (while keeping total IKKγ constant) results in increased type I IFN expression. Conversely, overexpressing IKKγΔ (in a background of constant IKKγ-WT expression) shows IKKγΔ functions as a dominant-negative IRF3 signaling inhibitor. IKKγΔ binds both IKK-α and β, but not TANK and IKKε, indicating that exon 5 encodes an essential TANK binding domain. Finally, IKKγΔ displaces IKKγWT from MAVS explaining its domainant negative effect.

**Conclusions/Significance:**

Relative endogenous IKKγΔ expression affects cellular selection of inflammatory/anti-viral pathway responses to ssRNA viral infection.

## Introduction

Activation of the mucosal innate immune response in sentinel epithelial cells is vital to the resolution of mucosal viral infection. Here, viral replication intermediates are sensed by cytoplasmic pattern recognition receptors, an event that activates two important signaling pathways, one mediated by the NF-κB transcription factor controlling inflammatory cytokine expression, and the second mediated by IRF3 controlling anti-viral type I IFN-α and -β expression. The coordinated expression of these two pathways is responsible for limiting viral replication and activating the adaptive immune response. Significant advances have been made in identifying the structure of these two pathways and their mechanism of control.

Cytoplasmic RNA virus infections, including Sendai (SeV)-, influenza-, Japanese encephalitis-, respiratory syncytial (RSV)- and others, produce 5'triphosphate modified- or ds-RNA products during their replication cycle. These “non-self” RNA species are bound by RIG-I, a cytoplasmic DExD/H box RNA helicase [1]–[3]. RNA-bound RIG-I is rapidly polyubiquitylated by E3 ligases (TRIM25 and Riplet/RNF13) that catalyze addition of Lys 63-linked ubiquitin polymers into the RIG-I·NH2 terminus [4], [5]. Lys 63-ubiquitinated RIG-I, in turn, associates with the mitochondrial antiviral signaling (MAVS) protein via its NH2 terminal caspase recruitment domain (CARD), producing an activated dimeric complex [6]. The assembled RIG-I·MAVS complex, in turn, recruits the TNF Receptor-associated factors (TRAFs)- 2, -3 and -6 to multiple TRAF-interaction motifs located in the MAVS proline rich domain [7]. This complex, serves as a scaffold for recruitment of signaling adapters mediating activation of the divergent NF-κB and IRF3 pathways. Downstream activation of the IRF3 pathway results in dramatic upregulation of RIG-I expression and signal amplification.

RIG-I·MAVS activates two distinct pathways controlling NF-κB, termed the canonical and cross-talk pathways. The canonical pathway is mediated by activating the IKK complex, a signaling complex containing the two closely related kinase subunits, IKKα and IKKβ, and a third regulatory subunit, IKKγ [8]. In the process of IKK activation, IKKγ is required for recruiting the catalytic IKK-α and β subunits to activated RIG-I·MAVS, where they are serine phosphorylated in their activation loops. IKK activation effects the phosphorylation and inducible degradation of the IκB inhibitor, resulting in nuclear translocation of the NF-κB/RelA transcriptional activator [9], [10]. Here, activated nuclear NF-κB induces expression of inflammatory cytokines such as Groβ, IL-6, IL-8 and others [11], [12]. By contrast, the cross-talk pathway is mediated by RIG-I·MAVS direct interacting with the IKKα-NF-κB inducing kinase (NIK) complex, in an IKKγ -independent manner [13]. This pathway, time-delayed relative to the canonical pathway, results in RelA and RelB release from cytoplasmic-sequestered p100. In this way RIG-I·MAVS induces two effector arms converging on NF-κB, producing mucosal inflammation.

RIG-I·MAVS also induces the IRF3 pathway, a pathway controlled by a complex of two IKK-related kinases, TANK-binding kinase 1 (TBK1) and an inducible subunit, IKKε [14]. Here, the TRAF-associated NF-κB activator (TANK) links TBK1and IKKε with upstream TRAF molecules [15], [16]. Importantly, IRF3 activation also requires the IKKγ signaling adapter; in IKKγ-deficient cells, IRF3 activation is also abolished in response to different RNA viruses [15]. As a result of IRF3 activation, the expression of type I IFNs results in a potent upregulation of RIG-I and its ubiquitin ligases, thereby potentiating coordinate signaling by the NF-κB and IRF3 innate signaling responses [17]. In this way, IKKγ serves as the final adaptor molecule in RIG-I·MAVS signaling that is shared between the canonical NF-κB and the IRF3 pathways.

In previous work, we identified an alternatively spliced IKKγ isoform, termed IKKγΔ. IKKγΔ is missing a crucial region in the NH2 terminal coiled coil domain whose functional effect is to couple IKK to distinct upstream signals. Interestingly, IKKγΔ efficiently mediates cytokine-induced canonical NF-κB activation by associating with the IKKα/β kinases, and mediates TAK/TAB and NIK inducible NF-κB activation, but is resistant to HTLV Tax [18]. Here we investigate its signaling role in response to ssRNA infection. In response to RSV infection, we find that IKKγ WT isoform is potently upregulated relative to the IKKγΔ splice form. In cells only expressing IKKγΔ, enhanced viral replication and cytopathic effect were seen due to deficient IRF3 signaling and type I IFN production. IKKγΔ functions as a dominant-negative inhibitor of IRF3 signaling being unable to couple to the TANK-IKKε complex and displaces IKKγ from activated RIG-I·MAVS. These data suggest that endogenous expression of IKKγD is involved in balancing inflammatory and anti-viral signaling response to ssRNA infection.

## Materials and Methods

### Cell Cultures

Human A549 pulmonary type II epithelial cells (American Type Culture Collection [ATCC]) were grown in F12K medium (Gibco) with 10% fetal bovine serum (FBS), penicillin (100 U/ml), and streptomycin (100 g/ml) at 37°C in a 5% CO2 incubator. Wild type and IKKγ^−/−^ [19] MEFs were cultured in Eagle's minimum essential medium (Gibco) with 0.1 mM nonessential amino acids, 1.0 mM sodium pyruvate, and 10% FBS. IKKγ and IKKγΔ reconstituated stable MEFs were described previously [18]. HEK293 cells were cultured in Eagle's minimum essential medium (Gibco) with 0.1 mM nonessential amino acids, 1.0 mM sodium pyruvate, and 10% FBS.

### Virus Preparation and Infection

The human RSV A2 strain was propogated in Hep2 cells and purified on sucrose cushion gradient [1]. Cells were infected at an MOI of 1.0 for indicated times. Sendai virus was purchased from Charles River Laboratory. Cells were infected with 100 hemagglutinin units/ml [20].

### Plasmid Construction

Expression vectors encoding Flag epitope-tagged RIG-I·NH2 terminus and Flag eiptope-tagged MAVS were described [21], [22]. pEF6-Flag-IKKα and pEF6-Flag-IKKβ were described in [18]. Myc-epitope tagged IKKγ and IKKγΔ were constructed by cloning PCR generated IKKγ cDNA into BamH1/HindIII sites of pcDNA3Myc. The PCR primers used were: 5′-ATCAATGGATCC ATGGAACAGAAGTTGATTTC CGAAGAAGAG CTCGGATCCATGAATTAGGCA CCT-3′ (upstream, Bam site underlined) and 5′-AGTATCAAGCTTCTACTC AATGCACTCC ATGACAT-3′ (downstream). pEGFP IKKγ and IKKγΔ were constructed by cloning the same cDNAs amplified using the same upstream primer and the downstream primer 5′-AGTATCAAGCTTCTC AATGCACTCC ATGACAT-3′ (to remove the stop codon) into Bam H1/HindIII digested pcDNA3EGFP, encoding a EGFP fusion on the COOH terminus of the IKKγ isoform. Plasmids were purified on Qiagen columns and sequenced for authenticity.

### Transfection

Two million freshly isolated cells were transfected in suspension with indicated plasmids according to the manufacturers recommendation (Amaxa). After transfection, cells were immediately transferred to DMEM and cultured for 24 h before treatment. Luciferase reporter assays were performed as previously described [23]. Data represents mean±SD of triplicate plates of normalized luciferase reporter activity.

### Quantitative Real-Time PCR (QRT-PCR)

Total RNA was extracted using acid guanidium phenol extraction (Tri Reagent; Sigma). For IFN and ISG analyses, 1 µgm of RNA was reversely transcribed using Super Script III in a 20 µl reaction mixture. One µl of cDNA product was diluted 1∶2, and 2 µL was amplified in a 20 µL reaction mixture containing 12.5 µL of SYBR Green Supermix (Bio-Rad) and 0.4 µM each of forward and reverse gene-specific primers (**Supplementary data, Table S1**), aliquoted into 96-well, 0.2-mm thin-wall PCR plates, and covered with optical-quality sealing tape. The plates were denatured for 90 s at 95°C and then subjected to 40 cycles of 15 s at 94°C, 60 s at 60°C, and 1 min at 72°C in iCycler (BioRAD). The IKKγ isoform specific QRT-PCR assays were performed in triplicate in an ABI Prism 7000 Sequence Detection System using the SYBR Green PCR Master Mix (ABI #4364344) as specified by the manufacturer. The final primer concentration was 900 nM (**Supplementary data, Table S1**). The PCR assays were denatured for 10 min at 95°C, followed by 40 cycles of 15 s at 95°C and 60 s at 60°C. After PCR was performed, PCR products were subjected to melting curve analysis to assure a single amplification product was produced. Quantification of changes in gene expression was using the ΔΔCt method using uninfected cells as a calibrator [1].

### Electrophoretic Mobility Shift Assay (EMSA)

A total of 35 µg whole cell extracts (WCEs) were incubated in DNA-binding buffer containing 5% glycerol, 12 mM HEPES, 80 mM NaCl, 5 mM DTT, 5 mM MgCl2, 0.5 mM EDTA, 1 µg of poly (dA-dT), and 100,000 cpm of ^32^P-labeled double-stranded oligonucleotide containing NF-κB binding sites [24] and IRF3 binding site [1] in a total volume of 25 µL. After fractionation in TBE acrylamide, gels were dried and exposed to BioMax film (Kodak) for autoradiography.

### Native PAGE for IRF-3 Dimer Formation

50 µg protein was fractionated by 7% native acrylamide gel in running buffer containing 1% sodium deoxycholate (Sigma Aldrich) as described [25]. After electrophoresis, proteins were transferred and analyzed by Western Immunoblot.

### Co-Immunoprecipitation and Western Immunoblot

Whole cell extracts (WCEs) were prepared using modified radioimmunoprecipitation assay (RIPA) buffer (50 mM Tris-HCl [pH 7.4], 150 mM NaCl, 1 mM EDTA, 0.25% sodium deoxycholate, 1% IGEPAL CA-630, 1 mM PMSF, 1 mM NaF, 1 mM Na3VO4, and 1 µg/ml each of aprotinin, leupeptin, and pepstatin). WCEs were pre-cleared with protein A-Sepharose 4B (Sigma) for 10 min at 4°C and immunoprecipitation was conducted for 2 hours at 4°C with primary Ab. Immune complexes were then precipitated by adding 50 µL of protein A-Sepharose beads (50% slurry) and incubated for 1 h at 4°C. Beads were washed three times with cold TB buffer (150 mM NaCl, 5 mM EDTA, 50 mM Tris-HCl [pH 7.4], 0.05% IGEPAL CA-630), and immune complexes were fractionated by 10% SDS-polyacrylamide gel electrophoresis and transferred to a polyvinylidene difluoride membrane by electroblotting. Membranes were blocked in 5% nonfat dry milk in Tris-buffered saline–0.1% Tween and probed with the indicated primary Ab. Membranes were washed and incubated with IRDye 700-conjugated anti-mouse Ab or IRDye 800-conjugated anti-rabbit Ab (Rockland, Inc.). Finally, the membranes were washed three times with TBS-T and imaged by an Odyssey infrared scanner. Sources of primary Ab were: anti-Flag M2 mAb (Stratagene), rabbit anti-IRF3 polyclonal Ab (Santa Cruz), anti-Myc mAb (Santa Cruz), anti-STAT1 polyclonal Ab(Santa Cruz) and anti-phospho-STAT1 (Cell Signaling).

### Confocal Immunofluorescence Microscopy

Transfected cells were plated on cover glasses pretreated with rat tail collagen (Roche Applied Sciences). After indicated stimulation, the cells were fixed with 4% paraformaldehyde in PBS and incubated with 0.1 M ammonium chloride (10 min). Cells were permeabilized with 0.5% Triton-100, followed by incubation in blocking buffer (5% goat serum, 0.1% triton X-100, 0.05% NaN3, and 1% BSA) and incubated with Anti-Rel A Ab (c-20, sc-372, Santa Cruz) in incubation buffer (0.1% triton X-100, 0.05% NaN3, and 2% BSA) overnight at 4°C. After washing, cells were stained with Alexa Fluor 555-conjugated Goat anti-rabbit IgG (Invitrogen) in incubation buffer for 1 h. After removing secondary antibody, the cells were fixed and counterstained with DRAQ5 (2 µM). The cells were visualized by Zeiss fluorescence LSM510 confocal microscope at 63X magnification.

## Results

### Selective IKKγ Expression in Response to ssRNA Infection

**Figure 1:**
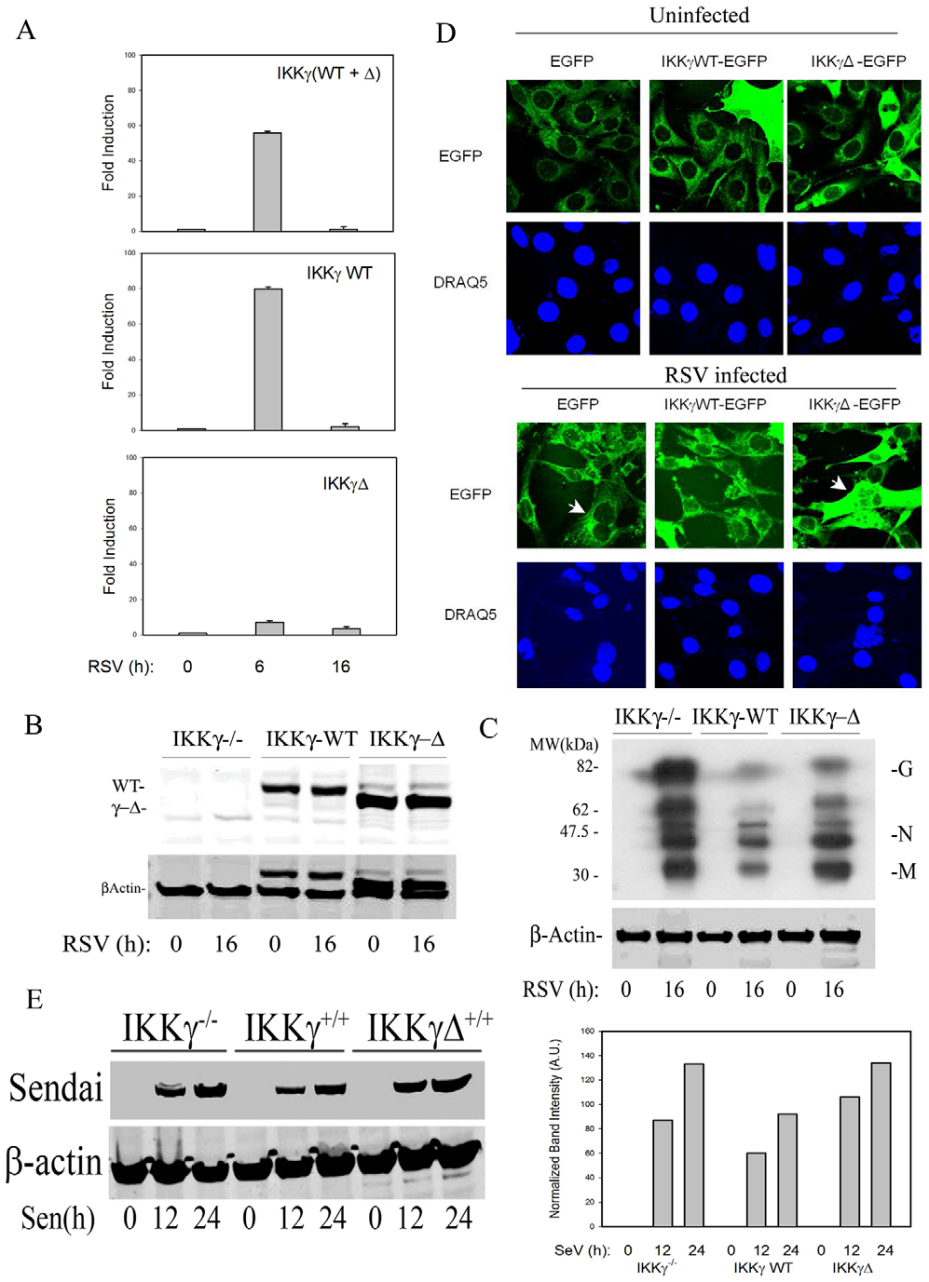
Enhanced viral protein expression in IKKγΔ reconstituted cells. (**A**) QRT-PCR of IKKγ isoforms in response to RSV infection. A549 cells were RSV infected (M.O.I. = 1) for indicated times, after which total RNA was extracted and assayed by QRT-PCR for IKKγ. Top panel, QRT-PCR for total IKKγ isoforms (using primers spanning Exon 2–3). Middle panel, QRT-PCR for IKKγ-WT isoform (using primers spanning the Exon 4–6 junction). Bottom panel, QRT-PCR for IKKγΔ isoform (using primers spanning the Exon 4–5 junction). Shown is fold change relative to uninfected cells. Data was reproduced twice, with similar results. (**B**) IKKγ^−/−^-deficient MEFs were reconstituted with empty vector (IKKγ^−/−^), IKKγ-WT or IKKγΔ and RSV infected (MOI = 1) for 0 or 16 h. 100 µγ whole cell extracts (WCEs) were assayed by Western immunoblot using anti-FLAG Ab. The locations of the two isoforms are shown. Bottom blot, the blot was reprobed with β-Actin Ab as a loading control (the FLAG bands are still visible). (**C**) Expression of RSV proteins were detected in WCEs using anti-pan RSV Ab (upper). β-Actin staining is used as a loading control (lower). (**D**) IKKγ^−/−^-deficient MEFs were transfected with expression vectors encoding EGFP, IKKg-WT-EGFP, or IKKgD-EGFP as indicated. Cells were then mock or RSV infected (M.O.I. = 1) for 24 h. Cells were fixed, stained with DRAQ5 (2 mM, Biostatus UK) and imaged by fluorescence microscopy in an LSM510 confocal microscope (magnification of 63X). Representative multinucleated cells contained within a single plasma membrane are indicated by white arrows. (**E**) IKKγ^−/−^-deficient MEFs reconstituted with empty vector (IKKγ^−/−^), IKKγ-WT or IKKγΔ were Sendai virus infected (MOI = 1) for 0, 12, 24 h. Protein expression was detected in WCEs using anti-Sendai Virus Ab (upper). β-Actin staining is used as a loading control (lower).

Previously we found that full length IKKγ-WT and the alternatively spliced IKKγΔ transcripts were expressed at 2∶1 ratios in uninfected A549 cells [18]. To determine whether IKKγ expression is affected by ssRNA virus infection, selective QRT-PCR assays were designed to measure total IKKγ isoform expression. Total IKKγ was quantified using primer pairs that selectively amplified the region corresponding to Exons 2–3; IKKγ-WT was quantified using primer pairs that selectively amplified the Exon 4-5 boundary; and IKKγΔ was quantified using primers that selectively amplified the Exon 4–6 boundary (Supplementary Table SI online). A549 cells were infected with sucrose cushion purified RSV, and IKKγ transcripts quantitated. Strikingly, relative to uninfected cells, total IKKγ transcripts were markedly induced 55- fold 6 h after RSV infection and returned to baseline 16 h later (Figure 1A, top panel). Similarly, full length IKKγ-WT was transiently induced 80-fold 6 h after RSV infection (Figure 1A, middle panel). By contrast, the IKKγΔ isoforms was only weakly (7-fold) induced by RSV infection at the same time point (Figure 1A, bottom panel). Together these data indicate that the differential expression of IKKγ splice forms is regulated by ssRNA infection.

### The Replication of RNA Viruses Is Increased in IKKγΔ Reconstituted MEFs

To selectively compare the function of IKKγ and IKKγΔ in response to RNA viruses, IKKγ^−/−^ mouse embryonic fibroblasts (MEFs) were transfected with full length IKKγ WT or IKKγΔ expression vectors and isoform expression quantified by Western immunoblot (Figure 1B). At equivalent amounts of expression vector, IKKγΔ expressed at a 2-fold higher level than did IKKγ-WT, and was not affected by RSV infection. To determine if IKKγ isoform expression affects RSV replication, the expression of RSV proteins were detected in Western immunoblot using a pan-anti-RSV Ab. As expected from their inability to produce type I IFN, RSV replicated to high levels in the IKKγ^−/−^ cells. In cells expressing IKKγ-WT, the level of RSV replication was significantly reduced (Figure 1C), consistent with the robust IFN production in RSV infected cells and the actions of type I IFN to restrict RSV replication [26]. By contrast, despite the findings that ectopic IKKγΔ had a slightly higher expression level than that of IKKγ-WT, there was a significant increase of RSV proteins produced 16 h after RSV infection (Figure 1C, compare G, N and M protein expression). Quantification of the RSV N protein by near-infrared scanning (LiCOR Odyssey) showed that the normalized abundance of N was 53 arbitrary units (AU) in IKKγ^−/−^ MEFs, 24 AU in IKKγ-WT expressing cells and 46 AU in IKKγΔ expressing cells. Similar findings were seen for RSV G and M proteins.

To confirm this result, multinucleated cell (MNC) formation, a consequence of RSV Fusion protein expression, was measured in IKKγ^−/−^ MEFs expressing either EGFP, EGFP-IKKγ-WT or EGFP-IKKγΔ [18]. MNCs were quantified by scoring 100 EGFP-positive cells in 5 randomly selected images by an observer blinded to the experimental condition. Twenty MNCs were observed in RSV-infected empty vector transfectants, whereas 9 were observed in EGFP-IKKγ-WT and 18 in EGFP-IKKγΔ transfectants. The reduction in MNC formation in IKKγ-WT transfectants is highly significant compared to empty vector transfectants (p<0.01, χ2 statistic ), whereas the number of MNCs in IKKγΔ was not different from empty vector. These data indicate that IKKγΔ is more permissive for RSV replication than IKKγ-WT. Similar findings were produced in IKKγ^−/−^ cells stably expressing IKKγ-WT, IKKγΔ or empty vector (Supplementary Figure S1 online).

We also investigated Sendai virus (SeV) replication in IKKγ-WT and IKKγΔ reconstituted IKKγ^−/−^ MEFs. Both at 12- and 24 h after infection, a significant increase of Sendai viral protein was observed in IKKγ^−/−^ and IKKγΔ reconstituted MEFs, as compared to those reconstituted with IKKγ-WT (Figure 1E). Quantification of the normalized protein abundance is shown in the adjacent graph (Figure 1E, right). Together these data suggested that IKKγΔ reconstituted MEFs were defective in restricting viral expression relative to those expressing the IKKγ-WT isoform.

### IKKγΔ Transfectants Are Defective in Type I IFN Production

**Figure 2:**
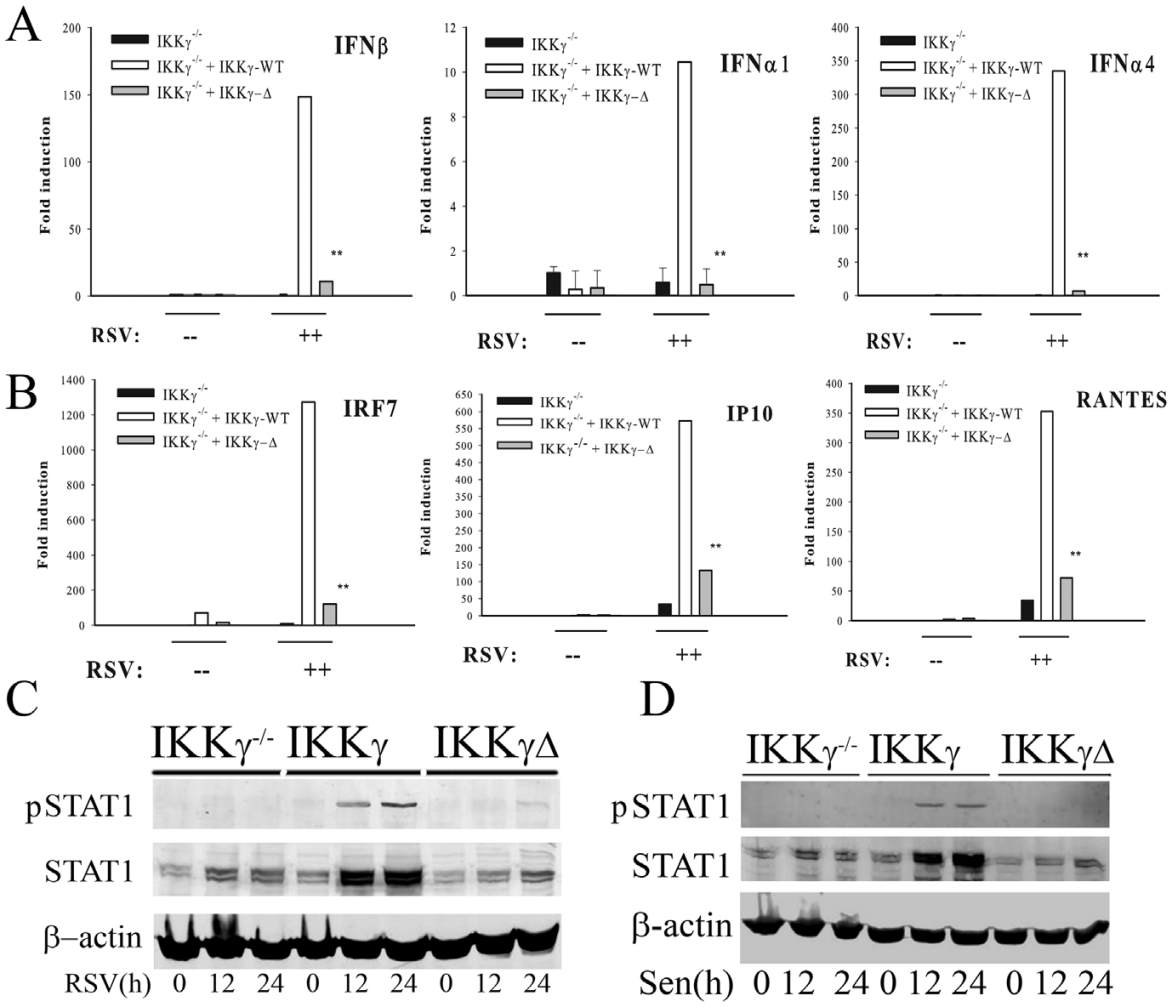
IKKγΔ does not mediate IFN or ISG gene expression in response to RNA virus infection. (**A**) IKKγ^−/−^ MEFs were reconstituted by empty vector, IKKγ-WT or IKKγΔ as indicated, and RSV infected for 16 h. Total RNA was extracted and QRT-PCR was conducted using probes for IFN-β, -α1, -α4. (**B**) Experiment as in (**A**) where QRT-PCR was was performed with probes for IRF7, IP10 and RANTES. (**C**) Experiment as in (**A**) where WCEs were extracted 0, 12 or 24 h after RSV infection and Western blot performed for phospho-Tyr^701^ STAT1 (pSTAT1, top panel) and total STAT1 (middle panel). β−actin was loading control (bottom panel). (**D**) Experiment as in (**C**) except cells were SeV infected for 0, 12 and 24 h.

To understand the mechanism for the enhanced viral replication rate in IKKγΔ-reconstituted MEFs, viral induced expression of type I IFN (IFN-β, -α1 and -α4), were quantified by QRT-PCR. In IKKγ^−/−^ MEFs, RSV infection did not induce a detectable change in expression of any type I IFN. Conversely, in cells reconstituted with IKKγ-WT, RSV induced a 150-fold increase in IFN-β, a 10-fold increase in IFN-α1 and a 350-fold increase in IFN-α4 (Figure 2A). Strikingly, in IKKγΔ-reconstituted cells, the expression of all three type I IFNs was significantly less, and for IFNα1, indistinguishable from that of IKKγ^−/−^ MEFs (Figure 2A).

A major mechanism for IFN induced antiviral activity involves the expression of downstream IFN-stimulated genes (ISGs). To confirm that the attenuated type I IFN production in IKKγΔ expressing cells was biologically relevant, we next measured ISG expression. In RSV infected cells reconstituted with IKKγ-WT, robust 1,200-fold induction of the IFN response factor-7 (IRF7), a 550-fold induction of IFN inducible gene -10 (IP10), and a 350-fold induction of RANTES were observed (Figure 2B). Conversely, in both IKKγ^−/−^ MEFs and those reconstituted with IKKγΔ, ISG expression was significantly reduced (Figure 2B).

To exclude the possibility that IKKγ reconstitution by transient transfection affects cellular signaling in response to ssRNA virus infection, the response of IKKγ^−/−^-deficient cells stably expressing IKKγ-WT and IKKγΔ, were investigated [18]. These cells have been previously shown to have intact NF-κB signaling in response to TNFα stimulation and IKKα/β expression [18]. In response to RSV infection, we found that these cells also had defective type I IFN expression (Supplementary Figure S2A online). Similar findings were produced in stable transfectants in response to SeV infection, both in terms of defective type I IFN production as well as impaired ISG expression (Supplementary Figure S2B, S2C online).

A biological action of epithelial type I IFN production is to induce paracrine activation of the Jak-STAT pathway in neighboring cells to produce a mucosal anti-viral state [27]. In this process, STAT1 is tyrosine phosphorylated and its expression upregulated via a positive feedback loop [28]. We therefore analyzed RSV-induced inducible STAT1 tyrosine phosphorylation and expression in IKKγ^−/−^ MEFs transfected with either empty-, IKKγ-WT and IKKγΔ expression vectors. The induction of phospho-Tyr^701^ STAT1 and upregulation of STAT1 protein were only observed in IKKγ-WT reconstituted cells (Figure 2C). Similar results were observed in response to SeV infection (Figure 2D). Collectively, these data suggest that, in contrast to IKKγ-WT, IKKγΔ does not effectively differently couple to type I IFN induction resulting in a deficient ISG response after ssRNA virus infection.

### IKKγΔ Is Deficient in Viral Induced IRF3 Activation

**Figure 3:**
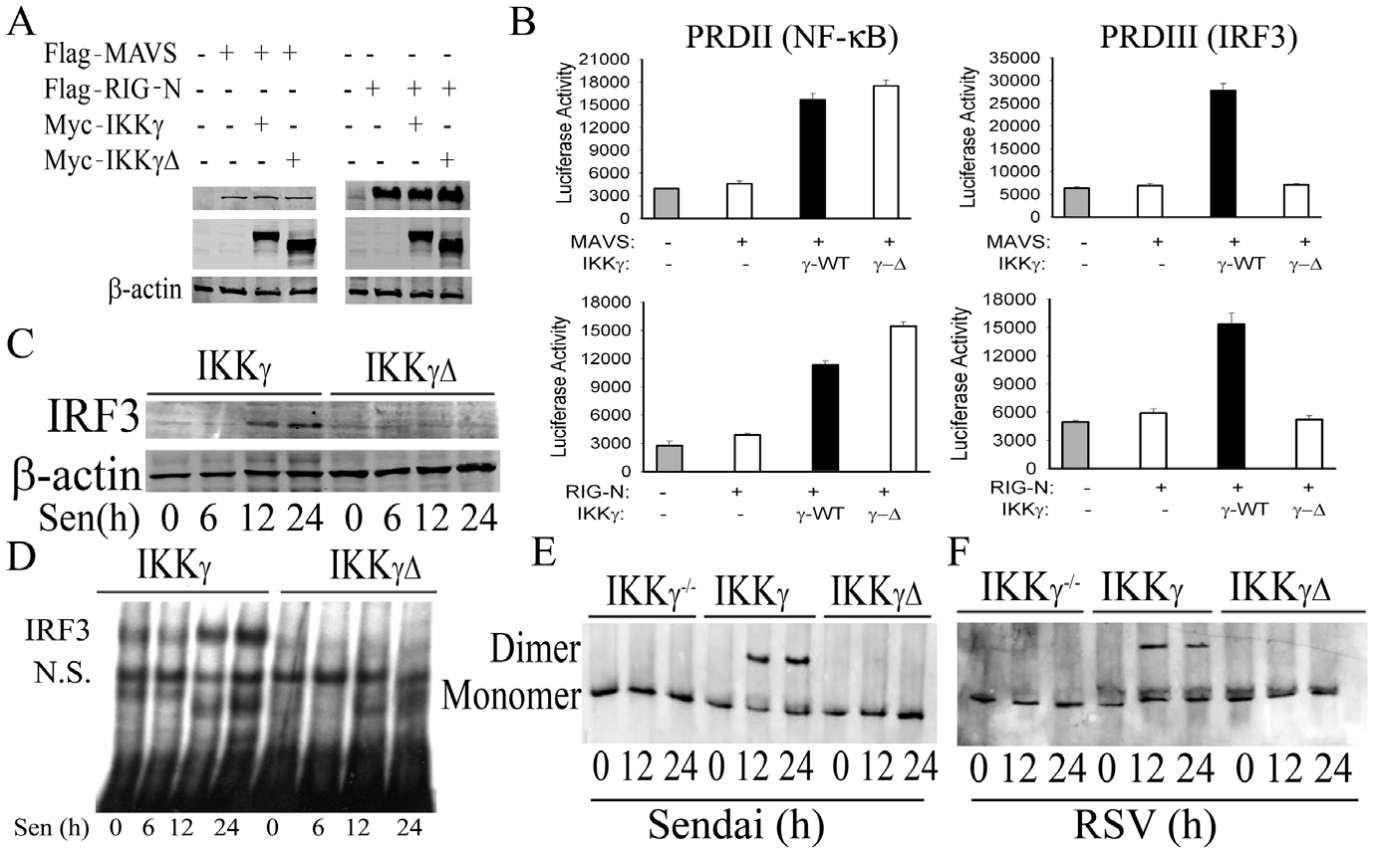
IKKγΔ is defective in mediating IRF3 signaling. (**A**) Expression vectors encoding Myc-tagged IKKγ or IKKγΔ were co-transfected into IKKγ^−/−^MEFs, in the presence of MAVS or NH2 terminal RIG-I (RIG-N). 48 h later, the expression of Flag-MAVS (top left panel), Flag-RIG-N (top right panel), Myc-IKKγ and Myc-IKKγΔ (middle panel) were detected by Western immunoblot. (**B**) IFN-β PRDII or PRDIII driven luciferase reporter genes were co-transfected with MAVS or RIG-N in the absence or presence of IKKγ and IKKγΔ as in (**A**). 36 h later, reporter activity was measured. Shown is normalized luciferase activity. (**C**) Nuclear extracts were prepared using sucrose-cushion from SeV infected IKKγ-WT and IKKγΔ stably transfected MEFs. The nuclei were denatured in 1% SDS PAGE loading buffer and IRF3 abundance measured by Western immunoblot. β-actin was used as loading control. (**D**) EMSA using an ISRE probe was conducted using nuclear extracts from a similar experiment as described in (**C**)**.** (**E,F**) IKKγ^−/−^, IKKγ reconstituted and IKKγΔ reconstituted MEFs were infected by SeV (**E**) or RSV (**F**) for times indicated. 50 µg WCEs were native gel-fractionated and Western immunoblot conducted using anti-IRF3 Ab. The location of monomer and dimers are indicated.

Previous studies have demonstrated that IKKγ is an essential adapter for IRF3 activation downstream of RIG-I·MAVS [15]. Because IRF3 is a major regulator of type I IFN production, we therefore tested whether IKKγ and IKKγΔ differentially affected viral induced IRF3 or NF-κB transcription. Myc epitope-tagged IKKγ and IKKγΔ were co-transfected in the absence or presence of MAVS along with NF-κB-selective (IFNβ PRDII domain) or IRF3-selective (PRDIII) luciferase reporter genes into IKKγ^−/−^ MEFs. MAVS expression was determined by anti-Flag Ab in Western Immunoblot (Figure 3A, left, top panel) and that of IKKγ and IKKγΔ by anti-Myc Ab in in Western immunoblot (Figure 3A, left, middle panel). We noted that IKKγ isoform expression did not affect MAVS expression.

As expected, MAVS was unable to activate NF-κB-driven luciferase reporter activity in IKKγ^−/−^ MEFs, and mediated a 4-fold increase in IKKγ-WT transfectants (Figure 3B). Importantly, MAVS activated NF-κB-driven luciferase reporter activity to a slightly greater degree (5-fold) in cells expressing IKKγ−Δ (Figure 3B, left, top panel). Conversely, although MAVS induced 3.5-fold increase in IRF3-driven luciferase reporter activity in cells expressing IKKγ-WT, no detectable induction of IRF3-driven luciferase activity was seen in cells expressing IKKγΔ (Figure 3B, right, top panel).

A similar experiment was performed with activated form of RIG-I, encoding the NH2 terminal CARD domain (RIG-N). Expression of RIG-N was determined by Western immunoblot (Figure 3A, top right), and that of co-transfected IKKγ isoforms (Figure 3A, middle right). Consistently with the findings of MAVS, RIG-N was unable to activate NF-κB-dependent reporter activity in IKKγ^−/−^ MEFs (Figure 3B, left, bottom panel) but did so in cells expressing either IKKγ-WT or IKKγΔ isoforms, where a 3-fold induction of PRDII was observed. Strikingly, and in a manner consistent with the expression of MAVS, RIG-N activated IRF3-dependent transcription only in the presence of IKKγ-WT, but was unable to activate IRF3 in IKKγΔ expressing cells (Figure 3B, right, bottom panel). Together these data indicated that IKKγ-WT mediates both NF-κB and IRF3 pathways, whereas IKKγΔ is unable to support IRF3 signaling.

To further define this mechanism, we examined whether IRF3 was induced to translocate into the nucleus in SeV-infected IKKγ^−/−^ deficient MEFs stably expressing IKKγ-WT or IKKγΔ. Sucrose cushion-purified nuclear extracts, free of cytoplasmic markers (tubulin [29]), were assayed by Western immunoblot using an anti-IRF3 Ab. In cells expressing IKKγ-WT, nuclear IRF3 was undetectable at 0- and 6 h, but appeared in the nuclear compartment after 12- and 24 h of SeV infection (Figure 3C). By contrast, in IKKγΔ expressing cells, no IRF3 was detected in the nucleus (Figure 3C), despite effective viral replication (Figure 1E). Next, nuclear extracts from SeV-infected MEFs were assayed for IRF3 DNA binding activity in EMSA using a radiolabeled ISRE site (taken from the ISG15 promoter). SeV induced a specific DNA binding activity 12- and 24 h after infection only in IKKγ-WT expressing cells; no DNA binding activity was seen in IKKγΔ expressing cells (Figure 3D; this band was previously shown to be DNA sequence specific and contain IRF3 [1]). To further confirm defective IRF3 activation, IRF3 dimer formation was quantified by Western immunoblot of native gel-fractionated whole cell extracts prepared from IKKγ^−/−^, IKKγ-WT-reconstituted and IKKγΔ-reconstituted MEFs infected for various times by either SeV or RSV. IRF3 dimer formation was detected only in IKKγ-WT- expressing cells in response to either type of viral infection (Figures 3E,F). We conclude that, in contrast to IKKγ-WT, IKKγΔ is unable to mediate IRF3 nuclear translocation, DNA binding, dimerization or transcriptional activation.

**Figure 4:**
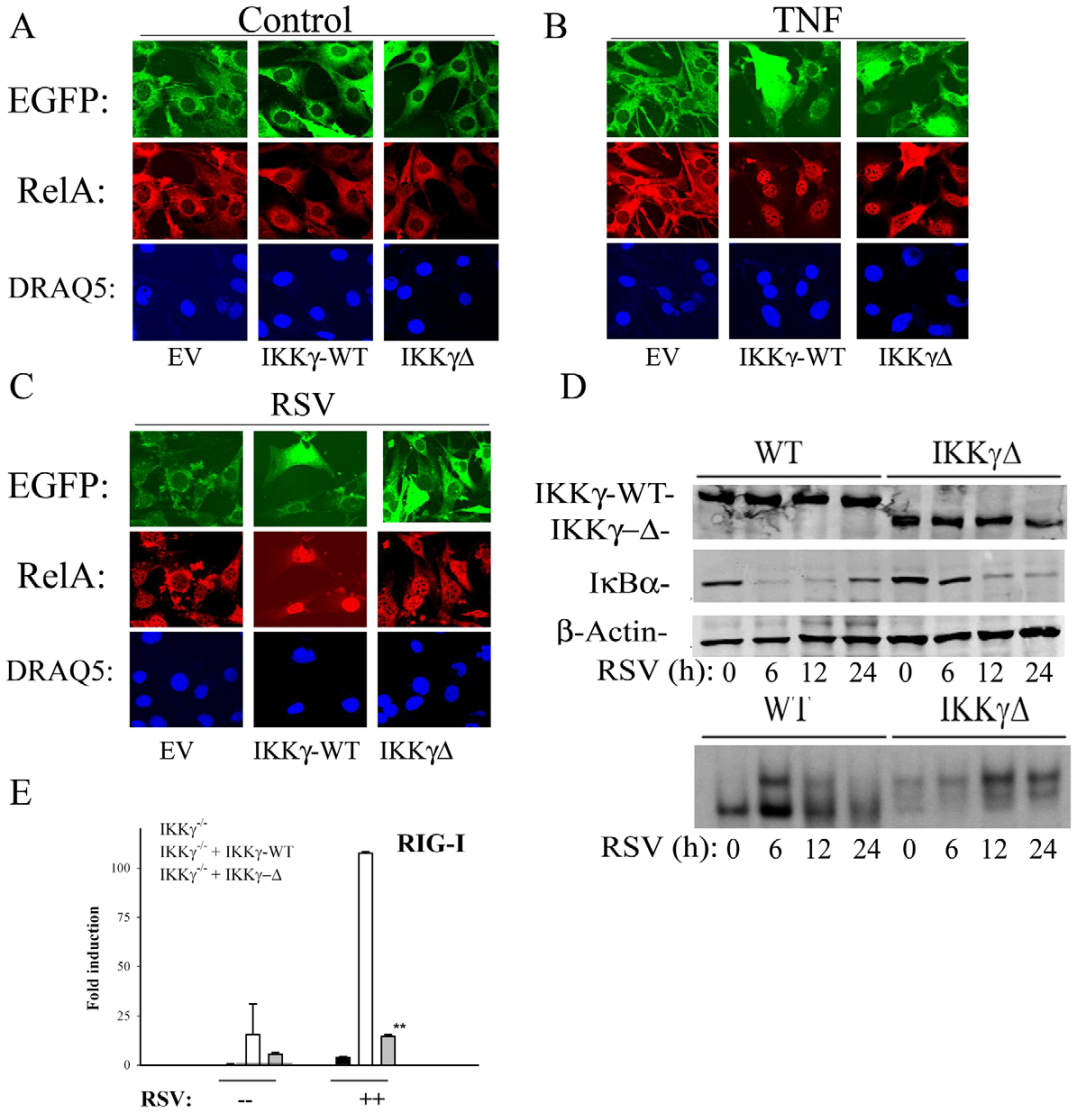
IKKγΔ couples to the canonical NF-κB activation pathway. (**A–C**) Confocal immunofluoresence microscopy. IKKγ^−/−^ MEFs were transfected with the indicated expression vectors encoding EGFP alone (EV), EGFP-IKKγ-WT or EGFP-IKK–γΔ. Cells were untreated, TNF stimulated (30 ng/ml, 1 h) or RSV infected (MOI 1, 24 h). Cells were fixed, stained with anti-RelA Ab and DAPI. Shown are relevant channels for EGFP, RelA and DAPI. (**D**) Stably transfected IKKγ-WT or IKKγΔ-expressing cells were RSV infected, and a time series of cytoplasmic and nuclear extracts prepared. Top Panel, Western blot for Flag (top), IκBα in cytoplasmic extracts. Bottom panel, EMSA using radiolabeled NF-κB probe. (**E**), IKKγ^−/−^ MEFs transfected with empty vector, IKKγ-WT or IKKγΔ were RSV infected for 16 h (MOI = 1). Total RNA was extracted and QRT-PCR was conducted using primers for RIG-I. **, P<0.01.

We next investigated whether IKKγΔ was coupled to the NF-κB pathway. First, confocal immunofluorescence experiments were performed for RelA nuclear translocation in IKKγ^−/−^ MEFs complemented with either EGFP-IKKγ or EGFP-IKKγΔ. Transfectants were then either treated with TNF (30 ng/ml, 1 h) or infected with RSV (MOI = 1, 24 h). Cells were fixed, RelA stained using anti-RelA Ab and transfectants imaged using confocal microscopy. Nuclei were counterstained with DAPI, and the presence of RelA examined in EGFP-expressing cells. In untreated controls, RelA was cytoplasmic in empty vector, IKKγ-WT or IKKγΔ expressing cells (Figure 4A). In response to TNF stimulation, a strong nuclear concentration of RelA was observed in either IKKγ-WT or IKKγΔ expressing cells but not those transfected with empty vector (Figure 4B). Similarly, RSV induced RelA nuclear translocation only in either IKKγ-WT and IKKγΔ expressing cells (Figure 4C).

A hallmark of the activated canonical NF-κB pathway involves cytoplasmic IκBα proteolysis via a ubiquitin proteasome-independent pathway [10], a phenomenon that is IKKγ-dependent [13]. To confirm that RSV-induced RelA nuclear translocation was mediated by canonical NF-κB pathway activation, IκBα proteolysis was measured in cytoplasmic extracts using Western immunoblot. In IKKγ-WT expressing cells, cytoplasmic IκBα proteolysis is clearly evident 6 h after RSV infection (Figure 4D), and is resynthesized 24 h after viral exposure via the RelA-IκBα positive feedback loop [30]. In IKKγΔ expressing cells, cytoplasmic IκBα proteolysis is also observed, although with slower kinetics. Conversely, in nuclear extracts, NF-κB DNA binding increases in IKKγ-WT-complemented cells 6 h after RSV infection, at times when cytoplasmic IκBα is degraded, and declines as IκBα is resynthesized, trapping NF-κB back in its cytoplasmic location (Figure 4D; supershifting experiments in RSV infected IKKγ^−/−^ MEFs have previously demonstrated this complex to be composed of RelA·p50 complexes[13]). Consistent with the qualitative differences in kinetics of IκBα proteolysis, NF-κB DNA binding increases in IKKγΔ-expressing cells, peaking at later times and persisting 24 h after infection (Figure 4D).

We sought to further understand the mechanism for qualitative difference in NF-κB activation in the IKKγΔ background. Previously we showed that RIG-I is strongly induced in response to RSV infection, and its expression is required for RSV inducible NF-κB activation [1]. Because RIG-I is type I IFN dependent, we examined whether RSV-induced RIG-I upregulation was attenuated in IKKγΔ-expressing cells by QRT-PCR. Although RIG-I mRNA is induced by over 100-fold in IKKγ-WT expressing cells, in both IKKγ^−/−^ and IKKγΔ expressing cells, viral inducible RIG-I expression was significantly reduced, accounting, in part, for the reduced NF-κB activation (Figure 4E). Together, these data indicate that the primary defect in IKKγΔ signaling is the IRF3 pathway, and the attenuated NF-κB activation is because of secondarily reduced RIG-I expression ( note ectopic RIG-I or MAVS expression can activate the canonical NF-κB pathway, seen in Figures 3A,B).

### Defective IRF3 Activation in Cells Expressing Increased IKKγΔ∶IKKγ-WT Ratios

**Figure 5:**
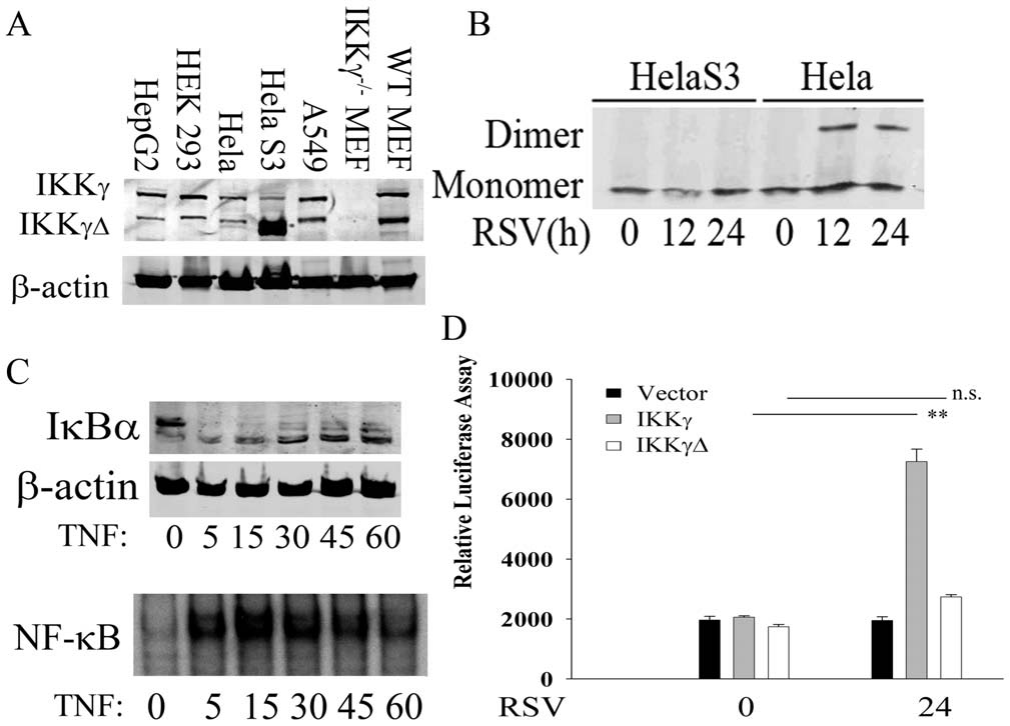
Defective IRF3 activation in cells expressing high endogenous levels of IKKγΔ. (**A**) 100 µg whole cell extracts (WCE) from HepG2, HEK293, Hela CCL3, Hela S3, A549, IKKγ^−/−^ MEFs and wild type MEFs were fractionated by SDS-PAGE and Western immunoblot was conducted using anti-IKKγ Ab. Location of IKKγ isoform is indicated. (**B**) Hela S3 cells were infected with RSV for 0, 12 or 24 h. WCEs were assayed for IRF3 dimerization using native gel electrophoresis. Shown is Western blot probed with anti-IRF3 Ab. Hela CCL2 cells were used as control. (**C**). Time course of TNF stimulation. Hela S3 cells were stimulated with TNF (30 ng/ml) for indicated times prior to cytoplasmic and nuclear protein extraction. Top, Western immunoblot using anti-IκBα Ab. β-actin staining is loading control. Bottom, NE were used in EMSA. Shown is a region of the autoradiogram with the specific NF-κB binding complex. (**D**) HelaS3 cells were co-transfected with the IRF3-dependent PRDIII luciferase reporter gene along with an empty vector, IKKγ-WT or IKKγΔ. 24 h after transfection, cells were infected with RSV for another 24 h. WCEs were collected and the luciferase assay was conducted. Shown is normalized luciferase reporter activity in triplicate independent plates. Data was reproduced in two independent experiments. n.s., not significant.

Previous work from our lab demonstrated that IKKγΔ was universally expressed in various ratios in different tissue- and cell types with IKKγ-WT [18]. To illustrate, the expression of IKKγ and IKKγΔ was surveyed in 7 different cell types by Western immunoblot, where the 43 kDa IKKγΔ and 50 kDa IKKγ-WT isoforms could be resolved. Both bands are specific as demonstrated by peptide competition experiments (Supplementary Figure S3 online). Both IKKγ and IKKγΔ isoforms are expressed from ∼2∶1 IKKγ∶IKKγΔ ratios in HepG2 or HEK293 cells, to 1∶2 IKKγ∶IKKγΔ ratios in WT MEF cells, to 1∶4 ratios in HeLa S3 cells (Figure 5A). We postulated that cells natively expressing high amounts of IKKγΔ could be defective in IRF3 activation. We therefore assayed IRF3 dimer formation in Hela S3 cells by Western immunoblot after native gel fractionation. Despite the finding that RSV replicated in Hela S3 better than that of Hela CCL2 cells (Supplementary Figure S4 online), HeLa S3 cells showed no evidence of IRF3 dimer formation. By contrast, efficient IRF3 dimer formation was observed in HeLa CCL2 cells (Figure 5B). To confirm HeLa cells could efficiently couple to the canonical NF-κB pathway, the response to TNF was measured. We observed that Hela S3 cells have an intact canonical NF-κB activation pathway indicated by rapid cytoplasmic IκBα proteolysis and appearance of nuclear NF-κB DNA binding activity in EMSA (Figure 5C these complexes have been extensively characterized by competition and supershift as containing RelA·p50 heterodimers [11], [31]).

To confirm that HeLa S3 cells had an otherwise intact RIG·MAVS-IRF3 pathway, HeLa S3 were complemented with IKKγ-WT. For this experiment, HeLa S3 cells were co-transfected with empty(pcDNA), IKKγ-WT, or IKKγΔ expression vectors and the IRF3-driven IFNβ PRDIII luciferase reporter gene. Cells were then RSV infected, and luciferase activity measured 24 h later. RSV was unable to activate PRDIII luciferase reporter activity in the cells transfected with empty vector or in those reconstituted with IKKγΔ (Figure 5D). However, RSV induced a 4-fold increase of IRF3-dependent reporter gene activity in the cells transfected with IKKγ-WT; by contrast empty vector-and IKKγΔ transfected cells did not induce IRF3 dependent transcription (Figure 5D). These data suggested IKKγ-WT complemented the IRF3 signaling defect in Hela S3 cells, cells selectively defective in IRF3 signaling but having an otherwise have an intact canonical NF-κB pathway.

### Varying Ratios of IKKγΔ (Keeping IKKγ Constant) Affects Viral Induced Type I IFN Production

**Figure 6:**
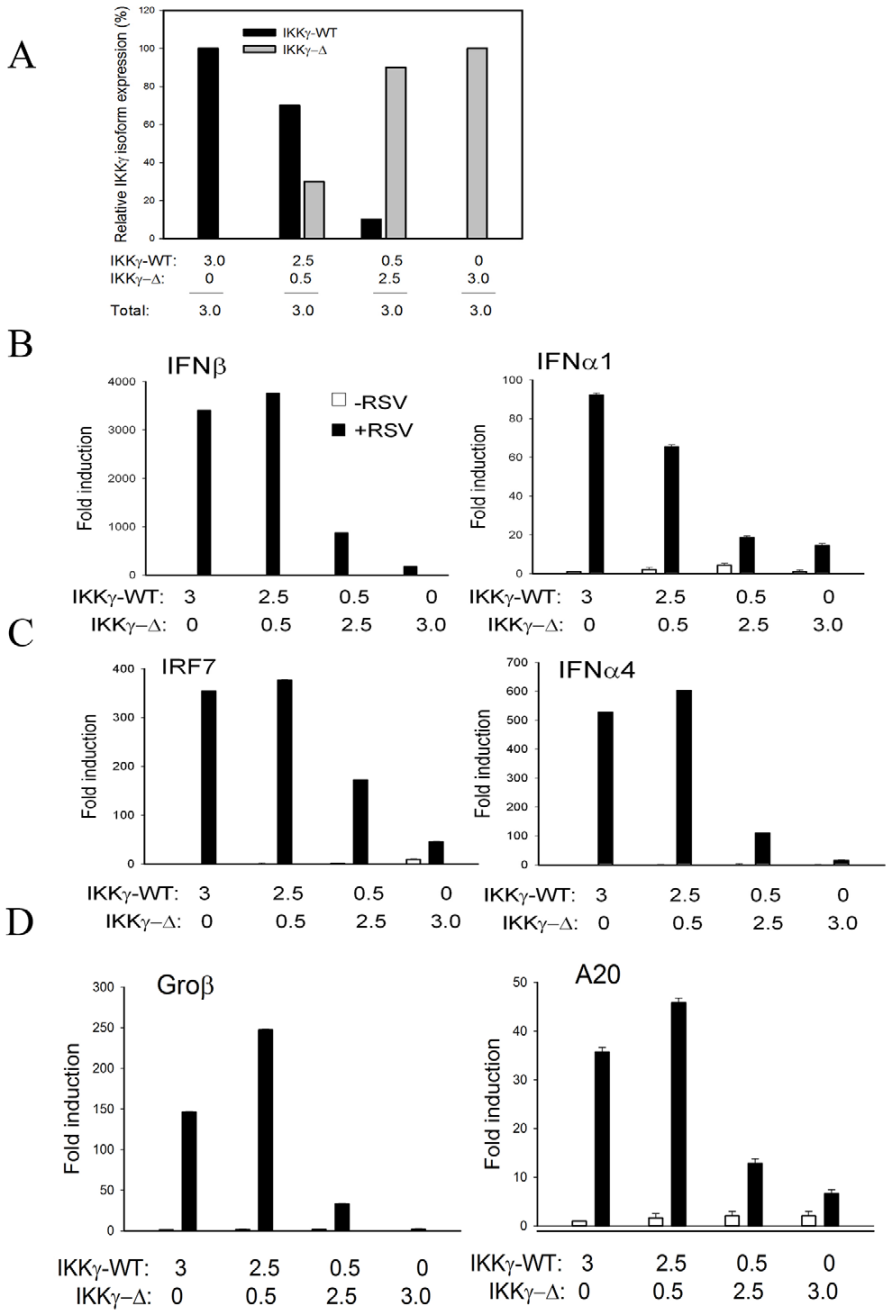
The IKKγΔ:IKKγ-WT ratio affects viral inducible type I IFN expression. (**A**) IKKγ^−/−^ MEFs were transfected with mixtures of eukaryotic expression vectors encoding IKKγ-WT, or IKKγΔ keeping the total IKKγ expression plasmid constant as indicated. Shown is quantitation of the Western immunoblot for the β-actin normalized expression for each isoform. (**B**) Effect on IFN expression. Transfectants were RSV infected for 0 or 16 h. Total cellular RNA was assayed by Q-RT-PCR for the expression of IFNβ, IFNα1, IRF7 or IFNα4 as indicated. Shown is X±SD of the fold mRNA induction relative to uninfected IKKγ-WT transfected cells. (**C**) Effect on NF-κB dependent gene expression. RNA was assayed by QRT-PCR for Groβ and A20 expression as indicated. Results were repeated three times with similar results.

Our findings in HeLa S3 cells suggested that the endogenous ratio of IKKγ-WT∶IKKγΔ is one determinant of type I IFN production in response to ssRNA virus infection. To more fully explore this hypothesis, we conducted an experiment varying the ratios of IKKγ-WT∶IKKγΔ in the IKKγ^−/−^ MEF background. In this experiment, we fixed the total amount of IKKγ to a constant level while only changing the IKKγWT∶IKKγΔ ratio (Figure 6A). Type I IFN production was then quantified in response to RSV infection using QRT-PCR. In cells expressing only IKKγ-WT, a 3,000-fold induction of IFNβ transcripts were observed in response to RSV infection, a response that was reduced in a dose-dependent manner upon the expression of IKKγΔ (Figure 6B). Under expression conditions where IKKγΔ was the predominant isoform, type I IFN production was significantly blunted (Figure 6B). Similar findings were observed for the ∼90-fold induction of IFNα1, 350-fold induction of IRF7 and 500-fold induction of IFNα4 mRNAs (Figure 6B). As expected from the blunted RIG-I induction in cells expressing IKKγΔ (see Figure 4E), the RSV inducible expression of NF-κB dependent Groβ and A20 genes were also blunted. Together we conclude that the relative IKKγ-WT∶IKKγΔ ratio, independent of changes in total IKKγ abundance, controls cellular IRF3-IFN responsiveness to ssRNA virus infection.

### IKKγΔ Overexpression Is a Dominant Negative Inhibitor of IKKγ-WT Mediated IFN Production

**Figure 7:**
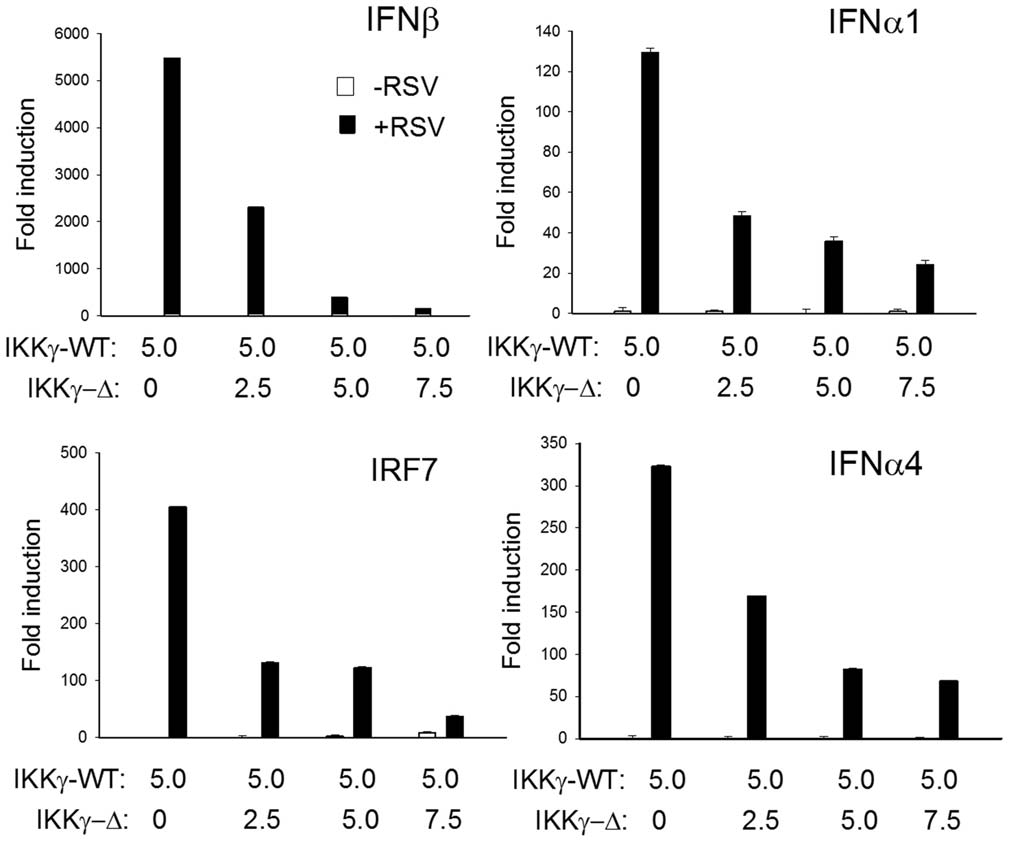
IKKγΔ is a dominant negative inhibitor of type I IFN expression. IKKγ^−/−^ MEFs were transfected with increasing amounts of IKKγΔ eukaryotic expression vectors in the presence of a constant amount of IKKγ-WT as indicated. 24 h later, transfectants were RSV infected for 16 h. Total cellular RNA was assayed by QRT-PCR for the expression of IFNβ, IFNα1, IRF7 or IFNα4 as indicated. Shown is X±SD of the fold mRNA induction relative to uninfected IKKγ-WT transfected cells. Results were repeated three times with similar results.

Earlier we showed that IKKγΔ is a dominant negative inhibitor of HLTV-I Tax induced NF-κB activation, despite the ability of both isoforms to bind HTLV-I Tax protein [18]. To explore whether IKKγΔ functioned as a dominant negative inhibitor of type I IFN production, we performed an alternative experiment where IKKγΔ was expressed in increasing amounts in the presence of a constant amount of IKKγ-WT in IKKγ^−/−^ MEFs (Figure 7). Type I IFN production was quantified in RSV using QRT-PCR. Co-transfection of IKKγΔ at 2.5 µg reduced type I IFN expression by ∼50% that was further reduced in a dose-dependent manner up to 7.5 µg expression plasmid, where the response was almost completely abolished.

Together we conclude that the relative IKKγ-WT∶IKKγΔ ratio primarily mediates IRF3-IFN responsiveness in response to RNA virus infection through its dominant negative effect.

### IKKγΔ Is Defective in Recruiting the TBK1 Adapter, TANK

**Figure 8:**
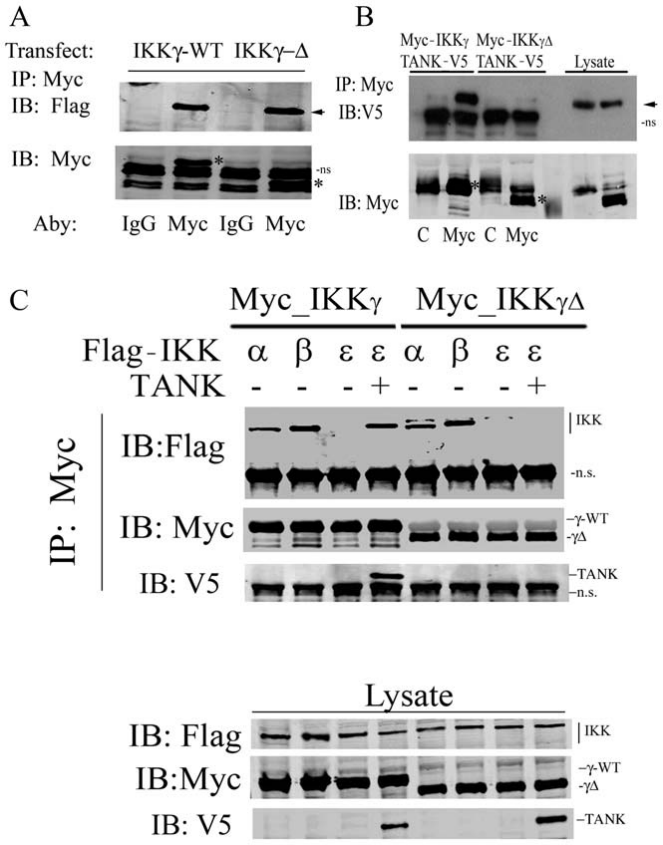
IKKγΔ does not interact with TANK·IKKε. (**A**) Myc epitope-tagged IKKγ or IKKγΔ was co-transfected with Flag-MAVS into HEK293 cells. 48 h later, 500 µg WCEs were collected and incubated with a control mouse IgG or anti-Myc Ab as indicated. Nondenaturing co-immunoprecipitation (IP) was performed using anti-Myc Ab; MAVS association was detected by Western immunoblot (IB) with anti-Flag Ab (top panel). Presence of Myc-IKKγ and Myc-IKKγΔ in the immunoprecipitates were detected by IB using anti-Myc Ab. Specific bands indicated by asterisks. ns, nonspecific band. (**B**) Myc epitope-tagged IKKγ or IKKγΔ was transfected into HEK293 cells with V5 labeled TANK. 48 h later, WCEs were immunoprecipitated with control (C) or anti-Myc Ab (Myc). TANK association was determined by IB using anti-V5 Ab. TANK is indicated by the black arrowhead. Bottom, the presence of Myc-IKKγ or IKKγΔ in immunoprecipitates were confirmed using anti-Myc Ab. Specific bands indicated by asterisks. (**C**) Flag epitoped-tagged IKKα, IKKβ or IKKε was co-transfected with Myc-IKKγ or Myc-IKKγΔ. IKKε was transfected in the absence or presence of TANK. 48 h later, WCEs were collected and subjected to nondenaturing co-immunoprecipitation using anti-Myc Ab. IKKα, IKKβ or IKKε association was detected by anti-Flag antibody (top panel). The presence of Myc-IKKγ, Myc-IKKγΔ and V5-TANK was demonstrated in the immune complexes by Western immunoblot. Expression of respective proteins was also confirmed in whole cell lysates using Western immunoblot (Lysate, bottom three panels). Location of specific bands are indicated at right.

Previous work has demonstrated that an interaction between the TBK1 adapter, TANK, and IKKγ is required for coupling RIG-I·MAVS complex to IRF3 activation [15]. Because IKKγΔ fails to activate IRF3, we first tested whether IKKγΔ associates with MAVS. For this purpose, Myc epitope-tagged IKKγ-WT or IKKγΔ expression vectors were co-transfected with Flag-tagged MAVS and subjected to nondenaturing coimmunoprecipitation using anti-Myc Ab. MAVS association was detected by immunoblot using anti-Flag Ab. We observed that both IKKγ and IKKγΔ effectively bound to MAVS (Figure 8A).

We next asked whether IKKγΔ was able to bind TANK. In this experiment, Myc epitope tagged IKKγ-WT or IKKγΔ expression vectors were co-transfected with V5-epitope tagged TANK. A nondenaturing co-immunoprecipitation assay experiment was then performed using anti-Myc Ab as the primary immunoprecipitating Ab, followed by Western immunoblot of the immunoprecipitates using anti-V5 Ab. V5-TANK was only observed in immunoprecipitates in cells expressing IKKγ-WT, but not IKKγΔ (Figure 8B, top panel). Equivalent amounts of IKKγ-WT and IKKγΔ were seen in the immunoprecipitates (Figure 8B, bottom panel).

TANK is an external adaptor that mediates the recruitment of the atypical IKK, IKKε, to IKKγ. Because IKKγΔ is unable to bind TANK, we investigated whether IKKγΔ was defective in IKKε recruitment. For this purpose, either Flag epitope tagged IKK-α, -β or -ε was co-transfected with Myc tagged IKKγ-WT or IKKγΔ and subjected to nondenaturing coimmunoprecipitation. To demonstrate the essential role of TANK for recruiting IKKε, V5-labeled TANK was also co-transfected with IKKε. After IKKγ isoforms were precipitated using anti-Myc Ab, the association of respective IKK was detected by anti-Flag Ab. Consistent with our previous work, IKKγ-WT associates with IKK-α and IKK-β [18]. IKKε did not bind to IKKγ-WT in the absence of TANK, but in cells cotransfected with TANK, IKKε could bind (Figure 8C, left panel). Conversely, although IKKγΔ bound IKKα and IKKβ, it did not recruit IKKε, even in the presence of TANK (Figure 8C, right panel). Based on these data, we conclude that IKKγΔ is unable to recruit TANK-IKKι to the activated RIG-I·MAVS complex.

### The Dominant Negative Effect of IKKγΔ Is Mediated by Displacement of IKKγ-WT from MAVS

**Figure 9:**
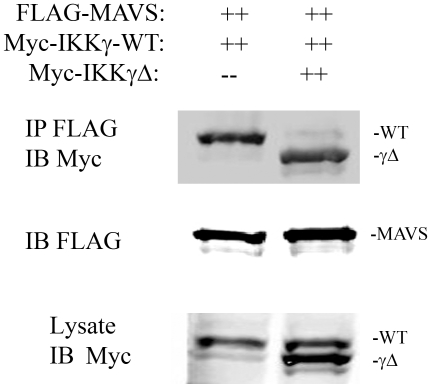
IKKγΔ competes with IKKγ-WT for MAVS binding. IKKγ^−/−^ MEFs were cotransfected with eukaryotic expression vectors encoding Myc-IKKγ-WT and FLAG-MAVS in the absence or presence of Myc-IKKγΔ. 36 h later, nondenaturing coimmunoprecipation experiments were conducted using anti-FLAG. Association of IKKγ isoforms were detected in the Western immunoblot using anti-Myc Ab.

To determine the mechanism for the inhibitory effect of IKKγΔ (Figure 7), we sought to determine whether IKKγΔ could displace the IRF3 signaling-competent IKKγ-WT isoform from the activated MAVS complex. For this experiment, Myc epitope tagged IKKγ-WT was co-transfected with FLAG-tagged MAVS in the absence or presence of IKKγΔ. Lysates were then subjected to nondenaturing coimmunoprecipitation using anti-FLAG, and IKKγ isoform association was determined by Western blot using anti-Myc Ab. In the absence of IKKγΔ, IKKγ-WT was associated with MAVS, however, the expression of IKKγΔ completely displaced IKKγ-WT from the MAVS complex (Figure 9).

## Discussion

IKKγ was identified as an essential regulatory subunit of the canonical IKK complex because IKKγ deficient cells were unable to activate NF-κB in response to most known stimuli [19], [32]. IKKγ plays multiple adapter roles in IKK activation through its ability to organize the assembly of IKKs into the activated high molecular weight complex [33], [34], bind ubiquitylated upstream signaling adapters [32], [35], [35]–[37], and recruit the IκBα inhibitor into the activated IKK complex [33]. Through these activities, IKKγ forms a molecular bridge between IKK, its upstream activators, and its substrate. In the innate immune response pathway, IKKγ recruits IKK−α and -β catalytic complexes to RIG-I·MAVS, resulting in IκBα proteolysis and canonical NF-κB activation. Similarly IKKγ is a binding target for TANK, an adapter that links TBK1 and IKKε, two key kinases controlling IRF3 activation [16], [38]. In this manner, IKKγ is the final common shared signaling adapter upstream of the divergent IRF3 and the canonical NF-κB pathways. Both IRF3 and NF-κB signaling play important, yet distinct, roles in anti-viral and inflammatory signaling in response to ssRNA viral infection. For example, IRF3 is a major mediator of type I IFN production, important in mucosal anti-viral response; in IKKγ^−/−^ cells, the replication of RNA viruses is significantly increased due to the inability to produce type I IFNs [15]. Similarly, NF-κB signaling is important in initiating mucosal inflammation and the adaptive immune response. The coordination and timing of these two arms of innate immune signaling response may affect the resolution of viral infection, yet the mechanisms for selection of these two pathways are not yet fully elucidated.

**Figure 10:**
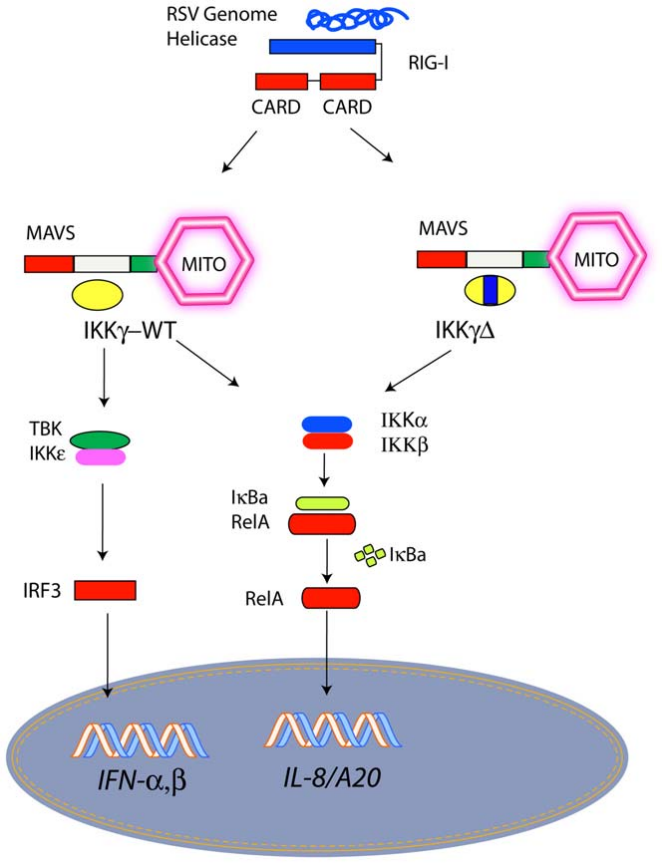
IKKγ-WT-IKKγΔ affects IRF3-NF-κB pathway utilization in ssRNA viral infection. Schematic diagram of two distinct signaling complexes produced by IKKγ splice variants. RIG-I recognition of ssRNA intermediates induces the activated RIG-IMAVS signaling complex. Depending on the relative abundance of IKKγ-WT and IKKγΔ isoforms, distinct signaling pathways are activated downstream. Mito, mitochondrion.

In this study, we have extended our previous work describing the signaling properties of a ubiquitously expressed IKKγ alternative splice product. Previously we reported that IKKγΔ is defective in mediating HTLV-Tax recruitment, but more efficiently mediates NF-κB activation by IKKα/β and MAP3Ks, NIK and TAK/TAB [18]. These earlier studies showed that IKKγΔ efficiently binds to IKKα/β isoforms in coimmunoprecipitation experiments, and induces IKK kinase activity to a greater degree than does IKKγ-WT. Here we find that IKKγΔ is primarily defective in IRF3 signaling, reducing type I IFN production and ISG signaling by displacing IKKγ-WT from MAVS complex with an isoform deficient in recruiting TANK-IKKε. Moreover, in cells naturally expressing high levels of endogenous IKKγΔ to IKKγ-WT ratios are defective in viral inducible IRF3 activation but respond via cytokine induced NF-κB activators. Strikingly, IKKγ-WT mRNA expression is highly inducible by ssRNA infection relative to IKKγΔ, suggesting that the cellular ratios of the two isoforms are dynamic. The mechanisms for this induction, and consequences in signaling will require further exploration. Together these findings indicate that relative endogenous expression of IKKγΔ isoforms may produce IKK complexes that differentially couple to distinct upstream signals, affording heterogeneity in cellular responses to otherwise similar activating stimuli (Figure 10).

IKKγ is encoded by a 10-exon-containing gene located at chromosome Xq28 [39]. Mutations in the IKKγ gene including different truncations of the IKKγ protein have been linked to the human syndromes of incontinentia pigmenti and anhidrotic ectodermal dysplasia associated with immunodeficiency [40]. Although human IKKγ transcripts containing an alternatively spliced first (noncoding) exon have been deposited in GenBank (AI24572, AF091453), these alternatively spliced transcripts encode wild type IKKγ [39]. IKKγΔ is the only alternative splice form known that affects the IKKγ coding region, and is caused by occlusion of exon 5. Using both 2D gel electrophoresis and a reverse transcription-PCR assay that distinguished the two isoforms, we found that IKKγΔ is widely expressed in normal human tissues in various relative ratios with fully spliced IKKγ-WT [18]. For example in normal breast and cervical tissue, IKKγΔ is the predominant isoform detected, whereas in normal liver and lung IKKγ-WT is predominant (HeLa S3, derived from a human cervical tumor maintains this IKKγΔ predominance). The findings that IKKγΔ expression reduces IRF3 signaling and type I IFN response in response to ssRNA virus infection may yield new insight for tissue-selective differences in anti-viral responses and tissue tropism in RNA virus infections [41].

We note that viral replication in IKKγΔ-expressing MEFs is enhanced relative to those cells expressing IKKγ-WT, but not to the degree seen in IKKγ^−/−^ MEFs (Figure 1C). In these experiments, viral inducible expression of the major type I IFNs, IFN-β, -α4 and -α1, is nearly absent and indistinguishable from that in IKKγ^−/−^ cells. That this degree of inhibition is biologically significant is demonstrated by the lack of detectable phospho-STAT formation or STAT autoregulation (Figure 2). It is surprising, then, that virus does not replicate in IKKγΔ−expressing cells to a similar degree as that seen in IKKγ^−/−^ cells. One interpretation of these findings is that IKKγΔ expressing cells, residual NF-κB activity may play a role in anti-viral response. In this regard, we note that others have suggested that NF-κB signaling mediates anti-viral activity [42]. Inhibition of NF-κB signaling in response to hPIV or RSV infection resulted in enhanced viral replication in an IFN-independent manner. The selective deficiency in IRF3 signaling in IKKγΔ expressing cells may allow the isolated study or identification of this potential anti-viral pathway.

The data in this study and that of our previous work have indicated that IKKγΔ is competent to mediate signals through the canonical NF-κB pathway. This conclusion is supported by multiple lines of evidence: 1. the ability of ectopic RIG-I or MAVS to activate NF-κB dependent gene expression in IKKγΔ expressing MEFs (Figure 3); 2. the ability of RSV infection to induce IκBα proteolysis in IKKγΔ expressing MEFs (Figure 3); 3. the ability of TNF to induce IκBα proteolysis in Hela S3 cells predominately expressing the IKKγΔ isoform (Figure 4); and 4. the ability of catalytic IKKα/β isoforms to bind IKKγΔ in nondenaturing coimmunoprecipitation assays (Figure 8). Previous protein interaction mapping studies that show the IKKα/β interaction motif lies in a 119 aa region in the far NH2 terminus of IKKγ [37], [43], [44], upstream of residues encoded by exon 5. Despite the ability to mediate productive IKKα/β binding and interaction, we note the qualitative difference in the kinetics of IκBα proteolysis in IKKγΔ expressing MEFs induced by RSV infection (Figure 4). Our data suggest that the slower kinetic response of NF-κB activation in IKKγΔ expressing cells is due to reduction in IRF3-IFN-RIG-I cross talk pathway.

Viral activation of RIG-I·MAVS signaling is thought to occur in two sequential phases. The first phase is mediated by low ambient concentrations of RIG-I early in the course of viral infection, where viral RNA is in low abundance [45]. Here, initial activation of IRF3 is mediated by the TRIM25 or Riplet/RNF135 ubiquitin ligases, inducing RIG-I ubiquitylation vis Lys 63- linked ubiquitin polymers, a modification that promotes its association with MAVS, initiating downstream IFN production [46]. The subsequent potent upregulation of RIG-I expression induced by this first wave of IFN [46] produces amplification of the signaling pathway. We observed that RSV-induced RIG-I upregulation is attenuated in IKKγΔ expressing cells, nearly to that seen in IKKγ^−/−^ MEFS (Figure 3). Taken in context with our earlier work showing that IFN-induced RIG-I upregulation is required for RSV-induced NF-κB activation [47], these data may suggest a mechanism for cross-talk between the IRF3 and NF-κB pathway in viral induced inflammation. In the absence of IRF3-IFN signaling, seen in IKKγΔ expressing cells, reduced RIG-I upregulation may result in delayed and or attenuated NF-κB activation.

Secondary structure predictions of IKKγΔ reveals that the occlusion of exon 5 affects the COOH terminus of an extended NH2 terminal coiled-coil motif at aa 174–224, a motif important in protein-protein interaction [18]. This alternative splice variant exhibits differential signaling by binding distinct signaling molecules. In this regard, IKKγ is essential for TNF signaling via its ability to recruit IKK to activated TNF receptors, mediate interaction with upstream MAP3Ks, and directly and/or recruit IκBα into the activated IKK for stimulus-induced phosphorylation. Recent work has shown that this scaffolding function of IKKγ may be due to its inducible post-translational modification via a unique chemistry of head-to-tail ubiquitin polymers catalyzed by the LUBAC ubiquitin ligase complex. These inducible ubiquitin polymers enhance the binding of upstream MAP3Ks, that phosphorylate associated catalytic IKKα/β subunits, resulting in their activation [36]. Interestingly, the LUBAC-mediated ubiquitin polymerization is involved in TNF inducible NF-κB signaling, but not by the related cytokine, IL-1. The role of post-translational modifications of IKKγ in response to ssRNA viral infection is not known. We note from co-immunoprecipitation experiments, that IKKγΔ associates with MAVS in the absence of ssRNA infection (Figure 8A), suggesting to us, that IKKγ-MAVS complex may be a stable, pre-formed complex whose activity is initiated by binding activated RIG-I.

Our findings that IKKγΔ displaces IKKγ-WT from the MAVS complex explains the dominant negative effect of IKKγΔ to reduce IRF3 activation. IKKγΔ is defective in TANK binding and consequently recruiting downstream TBK1·IKKι, a kinase complex essential in IRF3 phosphorylation. Like IKKγ, TANK is itself a scaffolding protein responsible for recruiting IKKε and TBK1 into an activated complex [38]. Structure-function studies of TANK have revealed a C2H2-type zinc finger in the TANK COOH terminus essential for IKKγ association [48]. Conversely, sequential mutagenesis and mapping studies on IKKγ have identified aa 150–250 aa as the TANK binding domain [15], [38]. Our study is consistent with these findings where IKKγΔ, lacking exon5-encoded 174–224 aa, is unable to bind TANK. In the absence of TANK binding, in IKKγΔ expressing cells, RIG·I MAVS is unable to signal to the IRF3 pathway, induce type I IFN expression or activate ISG signaling.

In summary, our findings reveal ubiquitously expressed IKKγ splice variant differentially couples IKK signaling to the IRF3 pathway and the induction of type I IFNs. Our studies further indicate that the relative level of expression of IKKγ splice forms affects IFN-mediated antiviral signaling in the host cell and may affect viral tissue tropism. Manipulation of the expression of these two isoforms may provide a mechanism to modulate the two arms of the innate immune pathway where preferential expression of NF-κB inflammatory signaling or IRF3 induced anti-viral signaling would be desired.

## Supporting Information

Table S1 Primers used for QRT-PCR
(5.05 MB TIF)

Figure S1 Enhanced cytopathic effect in IKKγ expressing cells. Wild type, empty vector, IKKγ-WT and IKKγΔ reconstituted IKKγ^−/−^-deficient MEFs were infected by RSV (M.O.I. = 1) for 24 h. Cells were also 4% paraformaldehyde fixed, stained with SYTOX (Molecular Probes) and imaged by fluorescence microscopy (magnification of 10X). Representative multinucleated cells are indicated by white arrows. In IKKγ^−/−^ or IKKγΔ,expressing cells, 13 and 15 multinucleated cells/high power field were detected respectively, while in wild type- and IKKγ-WT-reconstituted MEFs, only 1 and 2 fusion cells were observed.
(4.49 MB TIF)

Figure S2 Defective IFN response in cells stably transfected with IKKγΔ response to RNA virus infection. (a) WT MEFs, or IKKγ^−/−^ MEFs stably reconstituted with IKKγ-WT or IKKγΔ were RSV infected for 16 h (MOI = 1). Total RNA was extracted and QRT-PCR was conducted using probes for IFN-b, -α1, -α4. (b) WT MEFs, or IKKγ−/− MEFs stably reconstituted with IKKγ-WT or IKKγΔ were Sendai virus infected for 16 h. Total RNA was extracted and QRT-PCR was conducted using probes for IFN-b, and -α4. (c) Same experiment as in (b) where QRT-PCR was performed with probes for IRF7, IP10 and RANTES.
(5.61 MB TIF)

Figure S3 Antibody specificity. The staining specificity of anti-IKKγ Ab was evaluated using peptide preadsorption. Anti-IKKγ Ab was preadsorbed with nothing or 10-fold molar excess of recombinant purified GST-IKKγΔ-WT, and used as primary Ab in Western immunoblot. Both bands are reduced by 50%.
(0.19 MB TIF)

Figure S4 Effective RSV replication in HeLa S3 cells. Western immunoblot of HeLa S3 cells infected with RSV for indicated times.
(0.50 MB TIF)
