# Supplementary figures and images for: Expression of an IKKγ Splice Variant Determines IRF3 and Canonical NF-κB Pathway Utilization in ssRNA Virus Infection
Source: PLoS One. 2009 Nov 26;4(11):e8079. doi: 10.1371/journal.pone.0008079 (PMC2778955; doi:10.1371/journal.pone.0008079)

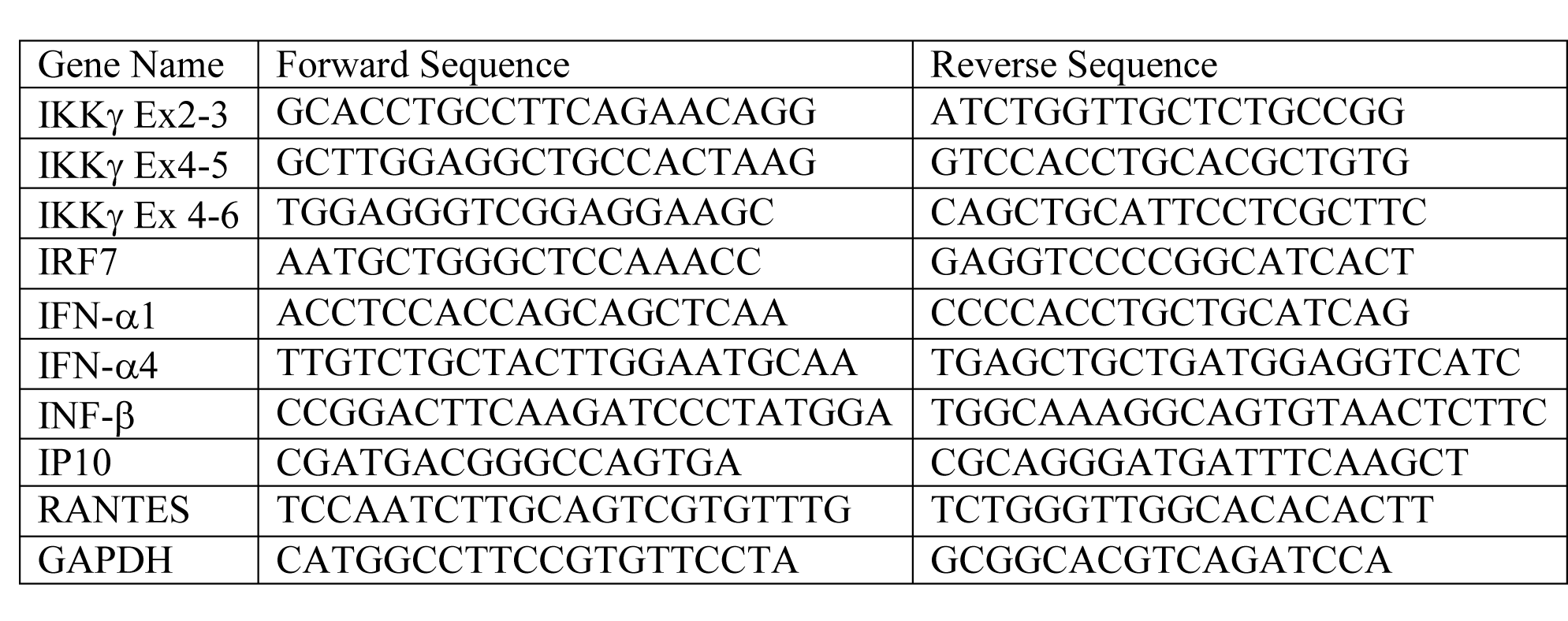

Supplement: Table S1 — Primers used for QRT-PCR (5.05 MB TIF) [file pone.0008079.s001.tif]

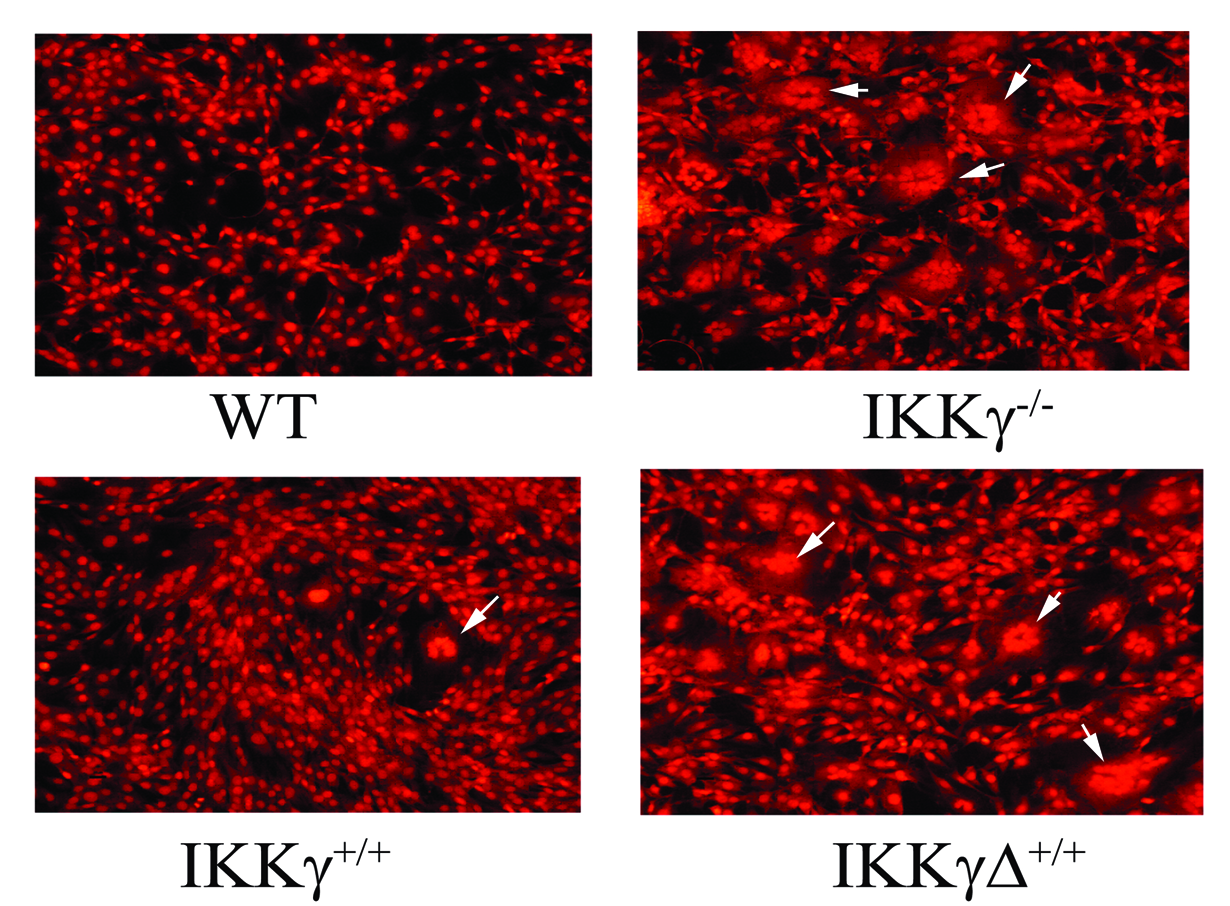

Supplement: Figure S1 — Enhanced cytopathic effect in IKKγ expressing cells. Wild type, empty vector, IKKγ-WT and IKKγΔ reconstituted IKKγ−/−-deficient MEFs were infected by RSV (M.O.I. = 1) for 24 h. Cells were also 4% paraformaldehyde fixed, stained with SYTOX (Molecular Probes) and imaged by fluorescence microscopy (magnification of 10X). Representative multinucleated cells are indicated by white arrows. In IKKγ−/− or IKKγΔ,expressing cells, 13 and 15 multinucleated cells/high power field were detected respectively, while in wild type- and IKKγ-WT-reconstituted MEFs, only 1 and 2 fusion cells were observed. (4.49 MB TIF) [file pone.0008079.s002.tif]

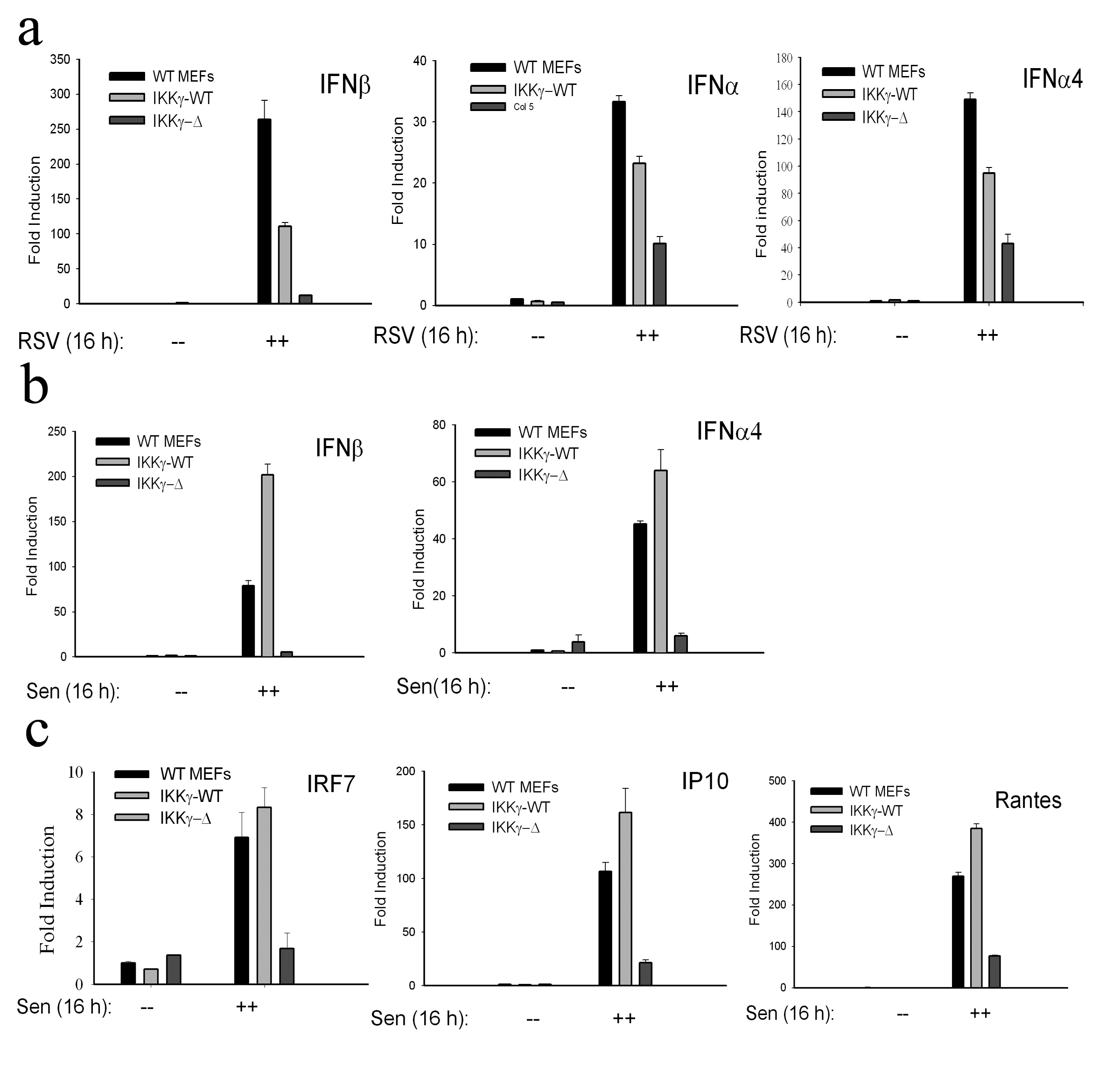

Supplement: Figure S2 — Defective IFN response in cells stably transfected with IKKγΔ response to RNA virus infection. (a) WT MEFs, or IKKγ−/− MEFs stably reconstituted with IKKγ-WT or IKKγΔ were RSV infected for 16 h (MOI = 1). Total RNA was extracted and QRT-PCR was conducted using probes for IFN-b, -α1, -α4. (b) WT MEFs, or IKKγ−/− MEFs stably reconstituted with IKKγ-WT or IKKγΔ were Sendai virus infected for 16 h. Total RNA was extracted and QRT-PCR was conducted using probes for IFN-b, and -α4. (c) Same experiment as in (b) where QRT-PCR was performed with probes for IRF7, IP10 and RANTES. (5.61 MB TIF) [file pone.0008079.s003.tif]

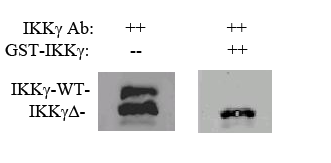

Supplement: Figure S3 — Antibody specificity. The staining specificity of anti-IKKγ Ab was evaluated using peptide preadsorption. Anti-IKKγ Ab was preadsorbed with nothing or 10-fold molar excess of recombinant purified GST-IKKγΔ-WT, and used as primary Ab in Western immunoblot. Both bands are reduced by 50%. (0.19 MB TIF) [file pone.0008079.s004.tif]

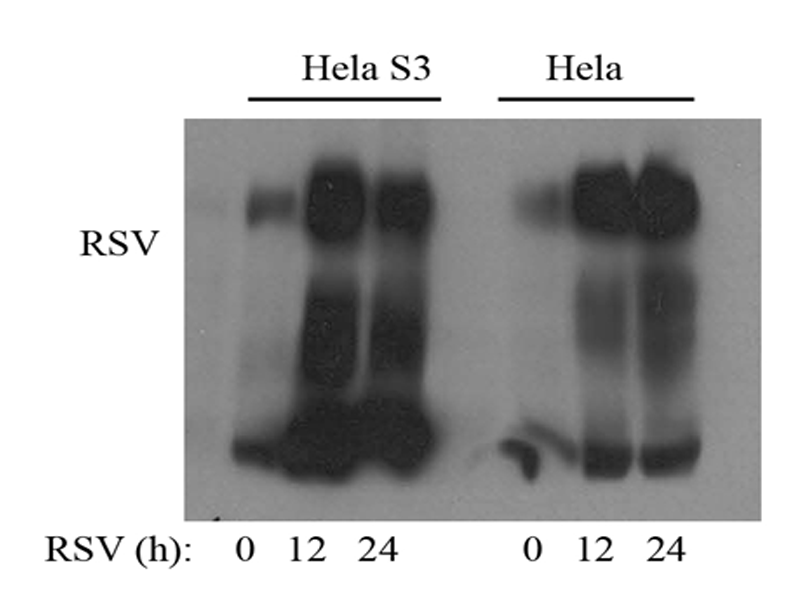

Supplement: Figure S4 — Effective RSV replication in HeLa S3 cells. Western immunoblot of HeLa S3 cells infected with RSV for indicated times. (0.50 MB TIF) [file pone.0008079.s005.tif]
